# Self-reported chronotype and objective sleep timing in university student athletes and non-athletes

**DOI:** 10.5935/1984-0063.20220062

**Published:** 2022

**Authors:** Kamila Litwic-Kaminska, Konrad S Jankowski

**Affiliations:** 1 Kazimierz Wielki University, Faculty of Psychology - Bydgoszcz - Kujawsko-Pomorskie - Poland; 2 University of Warsaw, Faculty of Psychology - Warsaw - Mazowieckie - Poland

**Keywords:** Actigraphy, Sleep, Circadian Rhythm, Students, Athletes

## Abstract

**Objective:**

The aim of this paper was to test how sport participation and chronotype affect objectively measured sleep timing parameters on workdays.

**Material and Methods:**

The sample included 82 student athletes and 40 non-athletes who completed three-day wrist actigraphy monitoring and the Polish version of the Morningness-Eveningness Questionnaire.

**Results:**

Eveningness predicted later timing of falling asleep and mid-sleep, but not the wake-up time. Student athletes had earlier wake-up time and shorter sleep duration than non-athletes.

**Discussion:**

The results support the view that university students suffer insufficient sleep, especially those participating in extensive sport activity.

## INTRODUCTION

Sleep plays an important role in physical and mental health, and is treated as a health factor^1^. Recommendations of the National Sleep Foundation advise that young adults at university student age (18-25 yrs.) should sleep around 7 to 9hrs^2^. The duration of sleep in many university students, however, is too low and problems with sleep are generally frequent. For instance, 25% of college students declared sleep duration less than 6.5hr per night^3^, while in the large cross-national study of Steptoe et al. (2006)^1^ university students were sleeping for around 7.5hr, and among them 21% had sleep duration of 6-7hr or less.

One of the factors affecting sleep duration in university students is chronotype^4^. This individual characteristic reflects differences between people regarding the time of day they prefer to sleep and undertake activities. Chronotype, also termed morningness-eveningness, is usually defined as a continuum between extreme morningness and extreme eveningness, with three chronotypes distinguished: the morning (‘larks’, M-type), the evening (‘owls’, E-type), and the neither type (N-type) in between them^5^. The M-type individuals, in contrast to the E-types, wake up and perform mentally and physically (including athletic performance) at their best in the earlier (morning) hours and they find it difficult to stay awake in the late-night hours^6^,^7^. Humans around the university students age are characterized by a tendency towards eveningness, which can cause decreased sleep duration if this clashes with the morning start of university activities^8^. Furthermore people participating in sports are known to exhibit greater morningness^9^.

Although physical activity is recognized as a factor improving the quality and quantity of sleep^10^, intensive training may also cause sleep disturbance^11^. Physical activity can help to recover after sleep disruption, but its effects depend on the timing of exercise^12^. Facer-Childs and Brandstaetter (2015)^7^ indicated that chronotype and wake-up time are significant determinants of sport performance. In their study, E-types, compared to M-types, needed more time after waking up to prepare for a sports activity.

Sufficient sleep is vital for proper functioning of all people, but especially for athletes due to its role in recovery and athletic performance^13^. University athletes, who need to combine the role of a student and an athlete, may be more likely to suffer from sleep problems resulting from the fact that, in comparison to their peers who are solely students, they carry more social roles and their daily obligations may not be in sync with their chronotype. Despite this, only two studies examining sleep timing parameters in university student athletes vs. non-athletes have been published so far: one using actigraphy monitoring^14^ and another using a sleep diary^15^. None of them, however, analysed the possible role of self-reported chronotype in the parameters, while Driller et al. (2017)^14^ pointed out the usefulness of chronotype questionnaires in athletes and non-athletes. Therefore, the aim of this paper was to test how sport participation and chronotype in conjunction affect objectively measured sleep timing parameters.

## MATERIAL AND METHODS

### Participants and procedure

The sample of 165 university students from [the name of the university has been removed to ensure anonymity for the authors of the study], consisted of two groups: student athletes and non-athletes. Participants started with filling out a questionnaire to collect data on sex and chronotype and verify the level of physical activity. Then, during three consecutive days (from Monday to Thursday, excluding holidays, vacations and exam sessions), the actigraphy measurements were employed to check the sleep parameters. The students were asked to wear the actigraph device continuously, except during training sessions or bath/shower time. In athletes, inclusion criteria determined a declaration of being involved in sports, understood as training at least three times a week in sports clubs, as a part of an academic sports association or having an individual training routine.

The majority of athletes were physical education students. Non-athlete students were from various fields of study. They were included if they declared a low level of physical activity and to be not engaged in any sport. Students whose physical activity level was assessed using a short version of the International Physical Activity Questionnaire (IPAQ) as high (despite earlier declarations of a low activity level) were eliminated. Exclusion criteria were health problems or diseases or taking drugs that might influence sleep parameters. Moreover, data from 27 athletes and 16 non-athletes were rejected from the analysis because the actigraphy device was worn for less than three nights. The final sample included 82 student athletes (22 females, aged 19-29, M=21.16, SD=1.94) and 40 non-athletes (27 females, aged 18-28, M=21.53, SD=1.97). The study was approved by the university ethics committee for scientific research.

## INSTRUMENTS

### Chronotype

The Polish version of the Morningness-Eveningness Questionnaire (MEQ^16^) prepared by Ciarkowska^17^ is a one-dimensional measure that allows the specification of a person’s sleep-wake cycle and the preferred hours of functioning. The higher the score, the more intensive the morningness preference.

### Physical activity level

The Polish version of the short IPAQ^18^ gathers information about the time spent sitting or walking as well as the time devoted to intensive and moderate physical activity within the previous seven days. A respondent evaluates the number of days within a week together with an average daily duration (in hours and minutes) devoted to physical activities that lasted continuously for at least 10 minutes without any significant breaks. By meeting particular criteria determined in the total weekly activity coefficient, calculated on the basis of the metabolic equivalent of work (MET) with the units expressed in MET • min/week, the subjects can be divided into three physical activity categories: high, moderate and low/insufficient. IPAQ was used to confirm low level of physical activity in the non-athletes group.

### Actigraphy

The Actiwatch AW4 (Cambridge Neurotechnology) and the Actiwatch 2 (Philips Respironics) actigraph wristwatches were used to monitor continuous movement activity (excluding the time of training, bathing and in circumstances in which physical damage to the equipment could occur). Before the research all the watches were calibrated and the subjects used the same devices in subsequent measurements. A 2-minute epoch length was applied. Sleep duration, mid-sleep, falling asleep and wake-up time were calculated.

### Statistical analyses

The analyses were performed in Statistica v. 13 (StatSoft, USA, Poland). The normal distribution of dependent variables was verified with Shapiro-Wilk’s test. Differences between student athletes and non-athletes were checked using Student’s t-test. Hedges’ g was used as effect size indicator. Pearson R correlation was applied to determine the relationship between all continuous variables. Next, analysis of covariance (ANCOVA) was conducted with group (athletes or non-athletes) and chronotype as independent variables and sex as a control variable. Sleep timing indicators from the actigraphy measurement were used as dependent variables.

## RESULTS

### Partial correlations

Chronotype was negatively correlated with mid-sleep and time of falling asleep and waking up, but did not correlate with sleep duration. Also, sleep variables were interrelated, with the exception of mid-sleep and sleep duration that were unrelated one to another (Table 1).

**Table 1:** Pearson R correlation matrix for the continuous variables.

Variables	Chronotype	Sleep duration	Fall-asleep time	Mid-sleep
**Sleep duration (h, min)**	0.10			
**Fall-asleep time (hh:mm)**	-0.34^***^	-0.46^***^		
**Mid-sleep (hh:mm)**	-0.33^***^	0.08	0.85^***^	
**Wake-up time (hh:mm)**	-0.22^**^	0.56^***^	0.47^***^	0.87^***^

Notes: Statistically significant associations:

** *p*=0.005,

*** *p*<0.001.

### Descriptive statistics

Student’s t-test indicates that athletes differ from the non-athletes in sleep duration, mid-sleep, and wake-up time (Table 2). Analyses of sex differences show that women sleep longer (t=-4.79, *p*<0.001, g=0.88; M_W_=6h 43 min, SD_W_=53 min; M_M_=5h 48 min, SD_M_=1h 07 min) and wake up later (t=-3.49, *p*=0.001, g=0.64; M_W_=06:59, SD_W_=01:10; M_M_=6:13, SD_M_=01:12) than men, making it important to include sex as a control variable. Males and females did not differ in chronotype.

**Table 2:** Differences between student athletes and non-athletes.

Variables	Athletes n=82	Non-athletes n=40	t	*p*	Hedges’ *g*
	M±SD	M±SD			
**Chronotype**	59.00±7.38	56.95±7.06	-1.46	0.147	0.28
**Sleep duration (h, min)**	5 h 55 m±1 h 9m	6 h 43 m±48 m	3.94	<0.001	0.76
**Fall-asleep time (hh:mm)**	00:21±01:07	00:21±01:08	0.01	0.990	0.00
**Mid-sleep (hh:mm)**	03:18±01:03	03:43±01:00	2.04	0.044	0.39
**Wake up time (hh:mm)**	06:16±01:16	07:04±01:00	3.53	0.001	0.68

### Sleep variable predictors

ANCOVA showed significant models for all of the studied sleep variables (Table 3). Sleep duration can be explained in 27%. Longer sleep was predicted by greater morningness scale and being non-athlete. Chronotype predicts 13% of fall-asleep time and 8% of mid-sleep variance. The fall-asleep time and mid-sleep of people with a greater tendency towards morningness occurs earlier. Thirteen percent of wake up time can be predicted by the group in such a way that non-athletes wake up later.

**Table 3:** Results of the ANCOVA predicting the actigraphy delivered sleep variables by chronotype and group, controlling for sex.

	ß	-95%; +95% CI	t	p
**Sleep duration: R^2^=0.27; F_(3,161)_=21.0, *p*<0.001**				
**Chronotype**	0.22	0.08; 0.35	3.18	0.002
**Group**	0.25	0.11; 0.40	3.37	0.001
**Fall-asleep time: R^2^=0.13; F_(3,161)_=9.07, *p*<0.001**				
**Chronotype**	-0.36	-0.51; -0.21	-4.82	<0.001
**Group**	-0.10	-0.26; 0.06	-1.23	0.222
**Mid-sleep: R^2^=0.08; F_(3,161)_=5.48, *p*=0.001**				
**Chronotype**	-0.28	-0.43; -0.13	-3.62	<0.001
**Group**	0.04	-0.13; 0.21	0.49	0.627
**Wake-up time: R^2^=0.13; F_(3,161)_=9.06, *p*<0.001**				
**Chronotype**	-0.12	-0.27; 0.03	-1.61	0.110
**Group**	0.16	0.00; 0.33	2.00	0.047

We used the following coding for groups: 1 = Athletes, 2 = Non-athletes; CI = Confidence interval for coefficients.

## DISCUSSION

Findings from this study are in line with previous reports^1^,^13^ and support the view that students suffer from insufficient sleep, which may have a negative impact on their health, well-being, and athletic performance. The students in both groups were falling asleep at a similar time, but the athletes were getting up earlier, and exhibited advanced midpoint of their sleep as a consequence. Our expectations that athletes sleep less have been confirmed. This is consistent with Driller’s actigraphy measurements^14^. Sleep duration of the athletes in our research was similar or shorter than in elite athletes^11^, what can be explained by the fact that we carried out measurements on weekdays only. According to Gupta et al. (2017)^11^ review, training days require getting up earlier what reduces sleep duration.

We found that athletes sleep less than non-athletes what is in contrast with indications that athletes require more sleep due to the recovery process^13^. In our study, we did not analyse subjective sleep quality what could have shown athletes’ satisfaction with their sleep. It is possible that owing to regular physical activity, it is easier for athletes to cope with sleep deficits, as Wolff and Esser (2019)^12^ pointed out. Nonetheless, earlier reports on the influence of sports activities on quality and quantity of sleep have not provided clear answer, indicating that there may exist differences related to the sports discipline^11^.

We did not find sex differences in chronotype in our sample. Some studies indicated that males are higher on eveningness than females, while others did not display sex differences^19^. The general conclusion is that sex differences in chronotype are rather weak and, therefore, require large samples to prove significant. Furthermore, factors such as the type of the questionnaire and culture may play a role; in a normalization study of MEQ in Poland there were no sex differences in chronotype in students’ age^17^. On the other hand, in our study women showed longer sleep than men, what is in line with results by Steptoe et al. (2006)^1^. Overall, based on age-adjusted cutoff points delivered from the Polish population norms of MEQ^17^ athletes and non-athletes exhibited average results indicative for N-types.

Collecting the results of sleep variable predictors together it can be stated that chronotype enables an explanation of how early or late a student is going to sleep, but the time when they wake up and the total time of sleep are rather connected with sport-engagement. Student athletes have to attend lectures and participate in sport activities at the university, which usually starts early in the morning. Some of student athletes’ also have obligatory morning workouts. These circumstances may not be conducive to people with a preference for eveningness who prefer to sleep longer^4^. This is important, given that shorter sleep durations were associated with higher levels of pre-training fatigue^11^ or higher risk of contusions^20^.

### Limitations

In the current study, we did not analyse the sleep timing parameters on free days. However, we were interested in assessing sleep in the weekdays during which students experience the greatest burden. It would be worth to consider factors affecting cognitive and physiological arousal, such as nutrition (e.g., consumption of caffeinated drinks), stress (prior to competition or in general), associated with sleep habits (e.g., sleeping place ­ dormitory or home) or with daily routines (time of sports activity, meals, and classes).

## CONCLUSION

An essential indication coming from the study for all students is to increase the amount of sleep, enhance sleep hygiene or at least create a nap strategy to fill the deficiency of the night sleep.
